# Comparative profiling of lactic acid bacteria isolates in optimized and spontaneous fermentation of cowpea leaves

**DOI:** 10.1002/fsn3.2140

**Published:** 2021-01-23

**Authors:** Joshua Ombaka Owade, George Ooko Abong', Michael Wandayi Okoth, Agnes Wakesho Mwang'ombe, Jared Omondi Jobor

**Affiliations:** ^1^ Department of Food Science, Nutrition and Technology University of Nairobi Nairobi Kenya; ^2^ Department of Plant Science and Crop Protection University of Nairobi Nairobi Kenya

**Keywords:** biochemical, cowpea leaves, fermented, pH, response surface methodology, titratable acidity

## Abstract

In as much as spontaneous fermentation of cowpea leaves enhances product diversification, the process is rather slow with poor product quality. Limited work has been undertaken to provide input toward standardization of the process and enhancing of product quality. The current study sought to evaluate the in‐process fermentative bacteria profile changes due to the effect of optimization of fermentation process of cowpea leaves. Lactic acid bacteria (LAB) isolates from spontaneous and optimized fermentation were characterized using biochemical tests, whereby optimization was done using the Response Surface Methodology model of the central composite design in the Design Expert Software. The RSM models accounted for 89% and 60% variability in the response variables of pH and titratable acidity, respectively (*p* < .001). Increasing the sugar concentration and period of fermentation significantly (*p* < .05) increased the titratable acidity, while reducing the pH. The optimal fermentation parameters were established as sugar and salt concentrations of 5% and 2%, respectively, 16 days of fermentation, pH of 3.8 and titratable acidity of 1.22% with a desirability of 0.859. Of the 13 identified LAB isolates, *Lactobacillus brevis* and *Lactococcus lactis* dominated the onset stage of spontaneous fermentation whereas only *Lactobacillus brevis* dominated the onset stage of optimized fermentation. Additionally, the final stage with the dominant isolates of *L. plantarum* was longer in the spontaneous fermentation process than in the optimized process. Evidently, optimizing the fermentation process resulted in increasing dominance by heterofermenters in the production of soured cowpea leaves, with the yielded product having enhanced acidity.

## Graphical abstract

Evaluation of acidity was a good indicator of fermentation process. Addition of sugar and salt improved the fermentation process of the cowpea leaves. Carbohydrate fermentation trends showed dominance of thirteen different LAB isolates.

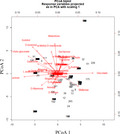

## 1 INTRODUCTION

Vegetable fermentation is an ancient practice that has over time gained importance in product diversification (Melini et al., 2019). The practice of vegetable fermentation was passed from generation to generation in the old times without full knowledge of the involved fermentative bacteria and the induced health promoting properties. Often, vegetables that were most abundant within the communities had such processing techniques employed in an effort to diversify their utilization. Recent developments in vegetable fermentation have promoted process optimization to the point of developing starter cultures from the most abundant lactic acid bacteria (LAB) isolates (Touret et al., 2018). This has aided technology transfer and commercialization of good quality products from the vegetables. Fermented vegetable products such as *Kimchi* and *sauerkraut* have been incorporated into diets and recipes of many countries through this (Özer & Kalkan‐Yıldırım, 2019). In improving keeping quality of the vegetables, the fermentation process often inhibits the growth of pathogenic and spoilage microorganisms (Khanna, 2019; Xiong et al., 2016). Through the hurdle technology, fermented vegetables subjected to drying can keep for three months, bridging the gap of seasonal availability of the vegetables (Muchoki et al., 2010). Moreover, the sensory quality which has often been a limiting factor in the continued utilization of especially the value added African leafy vegetables (ALVs) is improved (Ayed et al., 2020; Owade et al., 2019).

Fermentation of cowpea leaves has often been spontaneous; however, the limitation of this is the variability of the generated product and slowness of the process (Owade et al., 2019). Moreover, the key attribute in promoting consumer acceptance, sensory quality, in the product often tends to vary when such less optimized techniques are utilized in cowpea leaves fermentation. Since vegetables have been found to be low in fermentable sugars, Kasangi et al. (2010) recommended the addition of sugar at the levels of 1%–3%. In another study, while attempting to optimize the fermentation process of cowpea leaves, Muchoki (2007) similarly employed the one‐factor method in optimizing the sugar and salt concentrations; however, the limitation of the two studies was that they overlooked the interaction of the fermentation parameters being optimized. The proof of this is established through the higher pH and lower titratable acidity values recorded in the two studies compared to values reported in comparative studies on other fermented vegetables, 0.7%–1.54% for titratable acidity and a pH of 3.74–4.17 (Vatansever et al., 2017). Additionally, the need for optimization of salt concentration, which is of vital importance in controlling the growth of pathogenic and spoilage microorganisms like coliforms and yeast and molds during fermentation, results from the global move to control the immoderate use of salt as an ingredient in such processes (Khanna, 2019). The contribution of this study is to improve the low‐cost fermentation process of cowpea leaves existent in communities, while providing a case for commercialization of such products in effort to improve the utilization of the vegetables through value addition which is a major gap as of current (Owade et al., 2020). The work also forms the original basis to inform any possible food standards that would be developed for fermented cowpea leaves and other ALVs at large. Therefore, the study explored to characterize the microbial profile of the fermentative bacteria involved in the fermentation of cowpea leaves.

## 2 MATERIALS AND METHODS

### 2.1 Sample preparation

Kunde Mboga variety of cowpeas was grown at the field station of the University of Nairobi, Kenya, and the leaves harvested at eight weeks after emergence (WEA), which was the optimal stage of maturity as established by Owade et al. (2020), unpublished data. The harvested leaves were destalked to obtain the edible portion, washed, and shredded. The residual water from the washing was not drained for further use in the fermentation process.

### 2.2 Optimization of the fermentation process

Experimental runs were generated through the Response Surface Methodology (RSM) models of the Central Composite Design (CCD) of the Design Expert 11 software (StatEase, 2020); the illustrative formula is as shown in equation 1 (Behera et al., 2018). Three different fermentative factors were evaluated for optimization and they included concentrations of sugar and salt and the period of fermentation. The sugar and salt used in the study were the brown sugar (92.8% sucrose and 5.4% reducing sugars) and table salt (sodium chloride). The minimum and maximum entries of the factors were as used in similar studies by Muchoki (2007) and Kasangi et al. (2010), Table 1. Six center points and twenty experimental runs were generated in the study. Low‐cost fermentation was done for all the twenty experimental runs with the evaluation of response variables, pH, and titratable acidy (Table 2). Fermentation was done anaerobically with the vegetables placed in a bucket and submerged in the water, in which the generated ratios of salt and sugar dissolved. In order to prevent re‐entry of air, the buckets were covered with tightly fitting 500 gauge (125 micron) low density polyethene bag with continuous pressing of the vegetables to release the juice extract. Sampling of the fermentative solution for physico‐chemical evaluation was done as determined by the generated RSM ratios.

(1) $$N=2^{n}+2n+n_{c},$$

**TABLE 1:** The minimum and maximum levels of factors in the central composite design

Factor	Units	Minimum	Maximum	‐α	+ α
Concentration of salt	%	1	5	0.977311	6.02269
Concentration of sugar	%	1	5	0.977311	6.02269
Period of fermentation	Days	1	21	−4.11345	21.1134

**TABLE 2:** Central Composite Design for optimization of the fermentation process of cowpea leaves

Standard	Run	Factor 1: Salt Concentration	Factor 2: Sugar Concentration	Factor 3: Period of storage (days)	Response 1: pH	Response 2: Titratable acidity
6	1	5	2	16	4.71	0.423
4	2	5	5	1	4.63	0.2475
2	3	5	2	1	4.61	0.297
10	4	6.02	3.5	8.5	3.88	1.008
7	5	2	5	16	3.87	1.458
9	6	0.98	3.5	8.5	3.8	0.99
8	7	5	5	16	3.88	1.395
14	8	3.5	3.5	21.11	4.79	1.314
20	9	3.5	3.5	8.5	4.11	0.972
17	10	3.5	3.5	8.5	3.91	1.035
12	11	3.5	6.02	8.5	4	0.711
1	12	2	2	1	4.75	0.2565
13	13	3.5	3.5	0.11	6	0.288
18	14	3.5	3.5	8.5	4.44	0.711
16	15	3.5	3.5	8.5	3.59	0.459

Where *N* is the number of experimental runs, *n* is the number of factors, and *n_c_* is the number of central points generated.

### 2.3 Determination of optimal fermentation parameters

The multivariate design of experiment used in the study had pH and titratable acidity as response variables. The design of the experiment was set up as shown in equation 2 (Behera et al., 2018).

(2) $$y=f(x_{1},x_{2},x_{3})$$

Where *y* is the response variable, in this case either pH or titratable acidity, whereas *x*_(1‐3)_ are the independent variables concentrations of sugar and salt and period of fermentation.

The assumption of the design that both independent and response variables must be continuous was adhered to in the study. Randomization of the experimental variables was assumed to be achieved through the generated experimental runs. The predictor model for the response variables was generated using the second‐degree polynomial equation with the consideration of the full quadratic model coefficients and interaction factors as shown in Equation 3 (Arslan & Kara, 2017; Yabalak et al., 2019).

(3) $$y=a+\beta_{1}x_{1}+\beta_{2}x_{2}+\beta_{3}x_{3}+\beta_{4}x_{1}x_{2}+\beta_{5}x_{1}x_{3}+\beta_{6}x_{2}x_{3}+\beta_{7}x_{1}^{2}+\beta_{8}x_{2}^{2}+\beta_{9}x_{3}^{2}+\varepsilon ,$$

Where *y* is the response variable; is the constant coefficient; *x_1_*, *x_2,_* and *x_3_* are fermentation parameters to be optimized; *_1_*, *_2,_* and _3_ are linear coefficients; $\beta_{4}$, $\beta_{5,}$ and $\beta_{6}$ are coefficients of interaction factors; and $\beta_{7}$, $\beta_{8,}$ and are $\beta_{9}$ are coefficients of quadratic factors; and ε is the residual error.

### 2.4 Validation of optimal factors

Vegetables harvested at eight WAE, destalked, shredded, and washed were subjected to fermentation using the established optimal fermentation parameters. The pH and titratable acidity were evaluated based on the optimized fermentation period.

### 2.5 Culturing of LAB

Optimized and spontaneous (no sugar nor salt added) fermentation of cowpea leaves, each in duplicates, was done for vegetables harvested at 8 WAE. The vegetable were prepared as explained in the optimization process. The fermentative solution of the vegetables was sampled after every two days till the attainment of the optimal fermentation period. LAB cultures were plated on MRS (de Man, Rogosa, and Sharpe)‐agar plates as per ISO 15,214:1998 method (ISO, 1998a). The inoculated plates were incubated anaerobically (in anaerobic jars) at 30°C for 72 hr.

### 2.6 Isolation of LAB cultures from optimally fermented leaves

Acid producing colonies indicated by a clear zone around each of them were purified twice by replating in MRS agar plates with further incubation at 30°C for 72 hr each time. Only plates that numbered between 30 and 300 isolates were replated for the colonies were distinctively identified. Upon purification, the colonies were reselected and evaluated for catalase test and gram staining. Catalase‐negative and gram‐positive colonies were inoculated in stock solutions of 10% skim milk broth (w/v) and 20% glycerol (v/v). The stock solution was stored at −20°C for biochemical characterization within a period of two months.

### 2.7 Carbohydrate fermentation tests

A total of 267 microbial isolates (121 and 146 from optimized and spontaneous processes, respectively) were subjected to carbohydrate fermentation tests using the API 50 CHL strip for anaerobes for identification of the lactic acid bacteria following the manufacturer's instructions (BioMerieux, Lyon, France). The inoculation was done under aseptic conditions and the sugars incubated for 48 hr and recorded as either positive or negative. Trends of biochemical traits were drawn and the data matched with the API 50 CHL database in the catalogue by BioMerieux (2011).

### 2.8 Determination of pH and titratable acidity

The experimental runs and end products of spontaneous and optimally fermented cowpea leaves were tested for pH and titratable acidity. The titratable acidity was determined in duplicates as per the International Organization for Standardization (ISO) method 750:1998 (ISO, 1998b). Titratable acidity was calculated as g of lactic acid per 100g. The readings were determined in duplicate and the average recorded.

pH of the fermentative solution was determined as per the AOAC method number 981.12 (AOAC, 2005). The pH was determined using Ohaus model number ST2100, Ohaus Corporation USA. The pH meter was first calibrated with pH buffers of 4, 7, and 10. The fermentative solution was diluted 10 times with distilled water and the pH readings determined in duplicates and the average recorded.

### 2.9 Statistical analysis

The data for optimization of the fermentation parameters were analyzed using the analysis of variance (ANOVA) for quadratic models of the RSM models in the Design Expert version 11 software (StatEase, 2020). The statistical significance of the response model generated was tested using the *F* test in the same program. Effect of the independent factors on the response variables was generated using contour plots and 3‐D graphical display. The accuracy of the polynomial model that was generated was determined by the coefficient of R^2^. Statistical significance was tested at *p* < .05.

The data for the biochemical tests for the fermentative bacteria were analyzed using the R language for programming (R Core Team, 2019). The positive values were recorded as 1 and the negative as 0. Principal coordinate analysis (PCoA) of the biochemical traits was conducted to establish linkages and dissimilarities of the LAB isolates. The data were first standardized to normal distribution. Manhattan distance was used to achieve a better spread on the two dimensions. Dominance of the LAB cultures based on proportions and period of fermentation was generated over time.

## 3 RESULTS

### 3.1 Response surface methodology model for optimization of cowpea leaves fermentation

**FIGURE 1:**
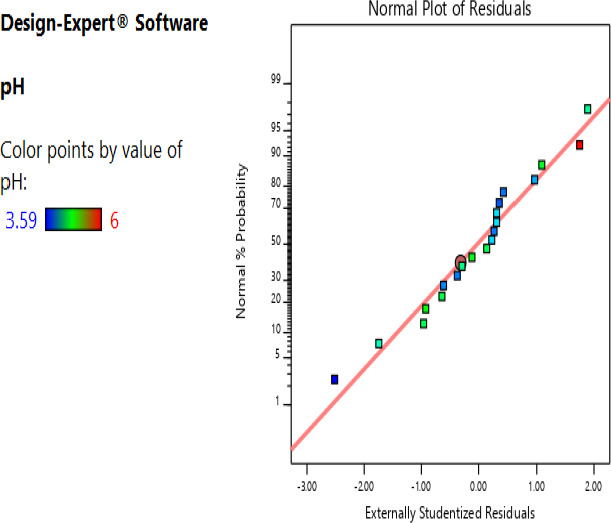
Studentized residuals and percent normality probability for pH

**FIGURE 2:**
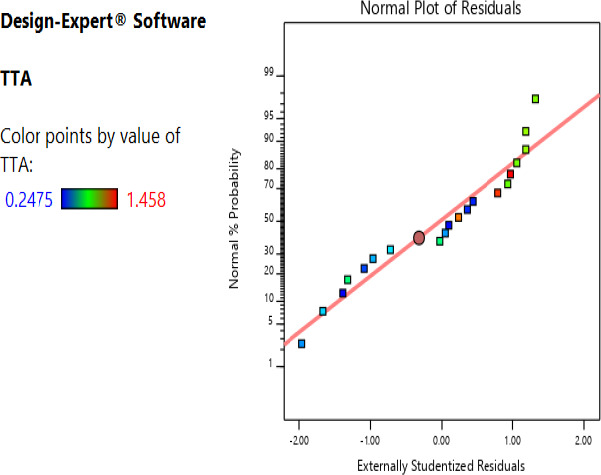
Studentized residuals and percent normality probability for titratable acidity

The adequacy of the distribution of the data to generate the predicted model was established by determining the normality; the data were found to have a satisfactory normality for the observed points clustered around the diagonal line (Figures 1 and 2).

**FIGURE 3:**
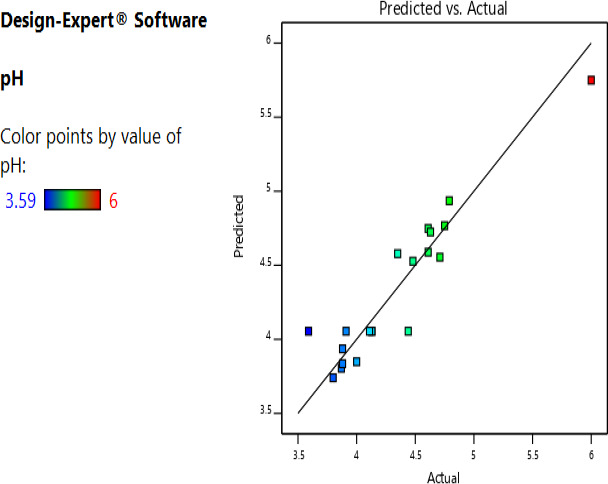
Actual and predicted values of pH of the fermented cowpea leaves

**FIGURE 4:**
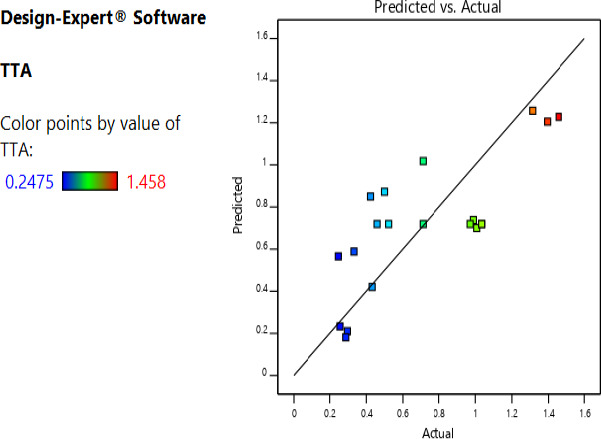
Actual and predicted values of titratable acidity of the fermented cowpea leaves

The actual and the predicted values for the pH and titratable acidity were as shown in Figures 3 and 4; the excellent distribution of the experimental points along the line of best of fit shows a good relationship between predicted and actual values. The predictive models for pH and titratable acidity were found to be significant (*p* < .01) with R^2^ of 0.885 and 0.60, respectively. The Model fitting the pH as a response variable had an *F*‐value of 8.56 implying that it only had a 0.12% for occurrence of residual error (noise). On the other hand, the model fitting titratable acidity had an *F*‐value of 7.98 with a chance of 0.18% of noise interfering with the model. The lack of fit of the predictive models of the pH and titratable acidity were not statistically significant (*p* > .05); the lack of fit of the two models occurring due to residual error was 72.7% and 45.7%, respectively. The coefficient estimates of the factors in the polynomial model were as shown in Table 3.

**TABLE 3:** Coefficient estimates of coded factors for pH and titratable acidity response variables

Factor	pH	Titratable acidity
Intercept	4.0500**	0.7193**
A‐Salt concentration	0.0282	−0.0113
B‐Sugar concentration	−0.2019**	0.1777*
C‐Period of fermentation	−0.2420**	0.3198***
AB	0.0412	Na
AC	−0.0038	Na
BC	−0.1488	Na
A^2^	−0.0943	Na
B^2^	0.0471	Na
C^2^	0.4555***	Na

Note

*significant at *p* < .05, **significant at *p* < .01, and *** significant at *p* < .001. na‐the constants were not generated.

### 3.2 Effect of concentration of sugar and salts and period of fermentation on pH and titratable acidity

**FIGURE 5:**
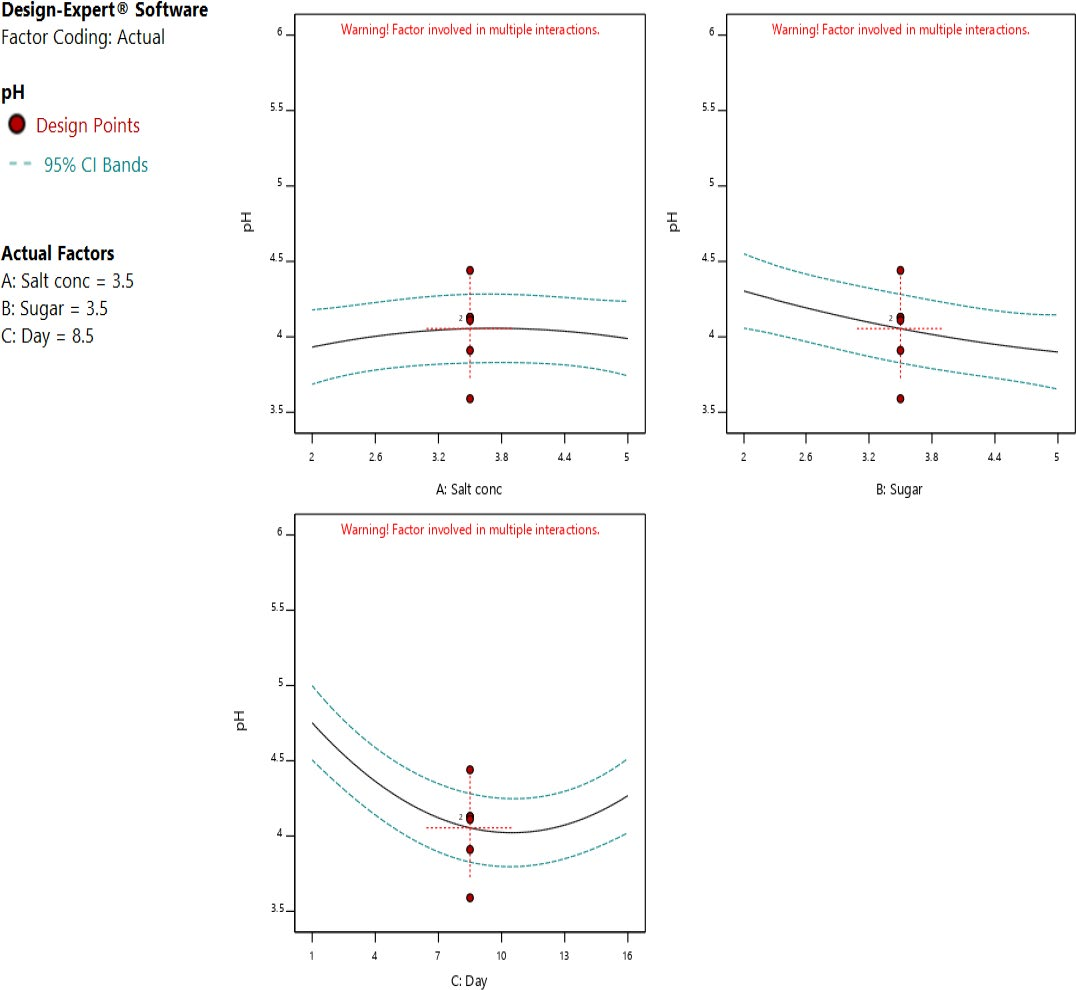
Effect of individual factors of concentrations of sugar and salt and period of fermentation on pH of fermented cowpea leaves

**FIGURE 6:**
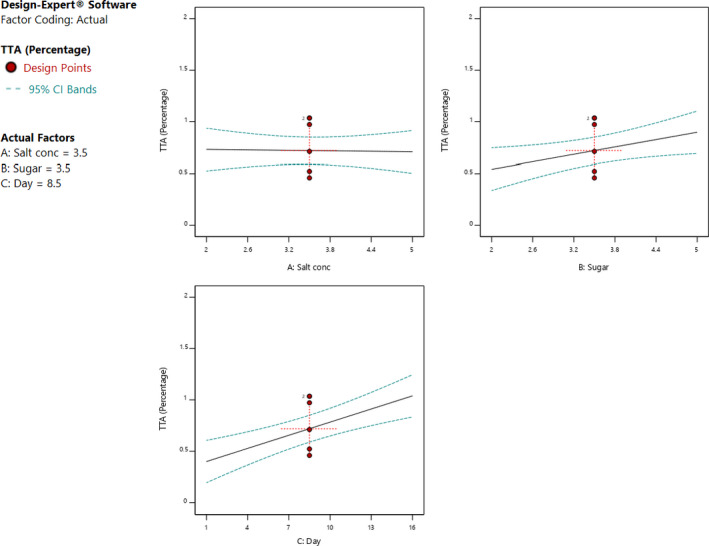
Effect of individual factors of concentrations of sugar and salt and period of fermentation on titatable acidity fermented cowpea leaves

**FIGURE 7:**
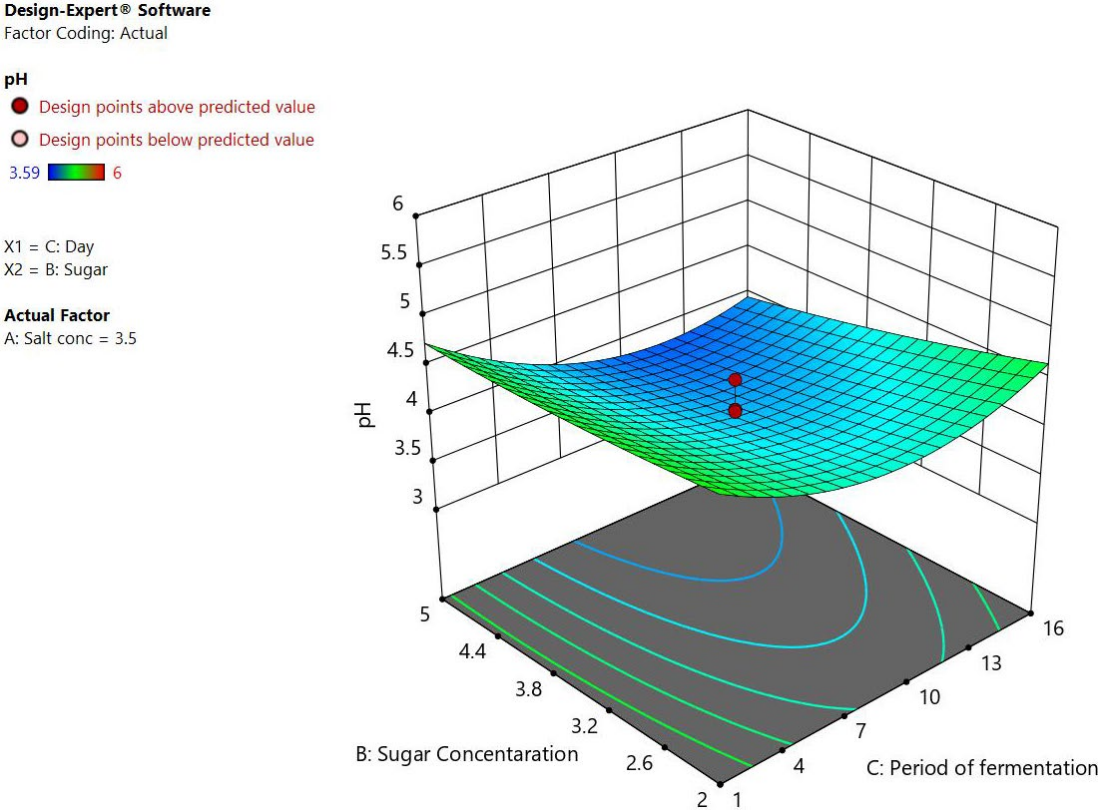
Three dimensional response surface plots showing the interactive effect of the concentrations of sugar and period of fermentation at 3.5% salt concentration

**FIGURE 8:**
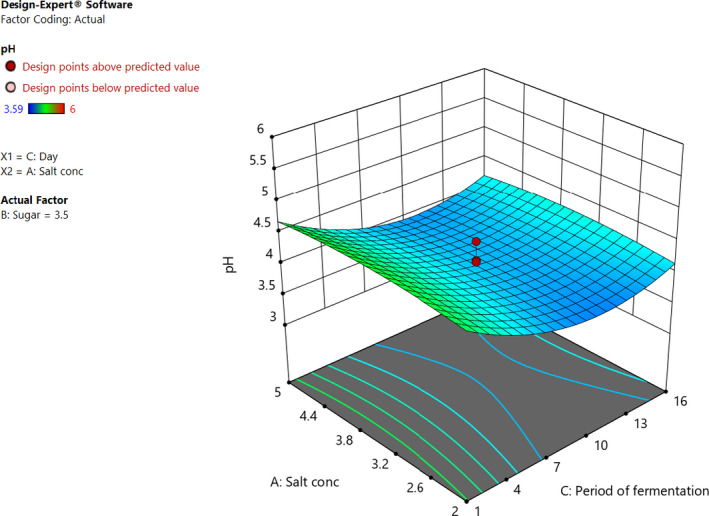
Three dimensional response surface plots showing the interactive effect of the concentrations of salt and period of fermentation at 3.5% sugar concentration

**FIGURE 9:**
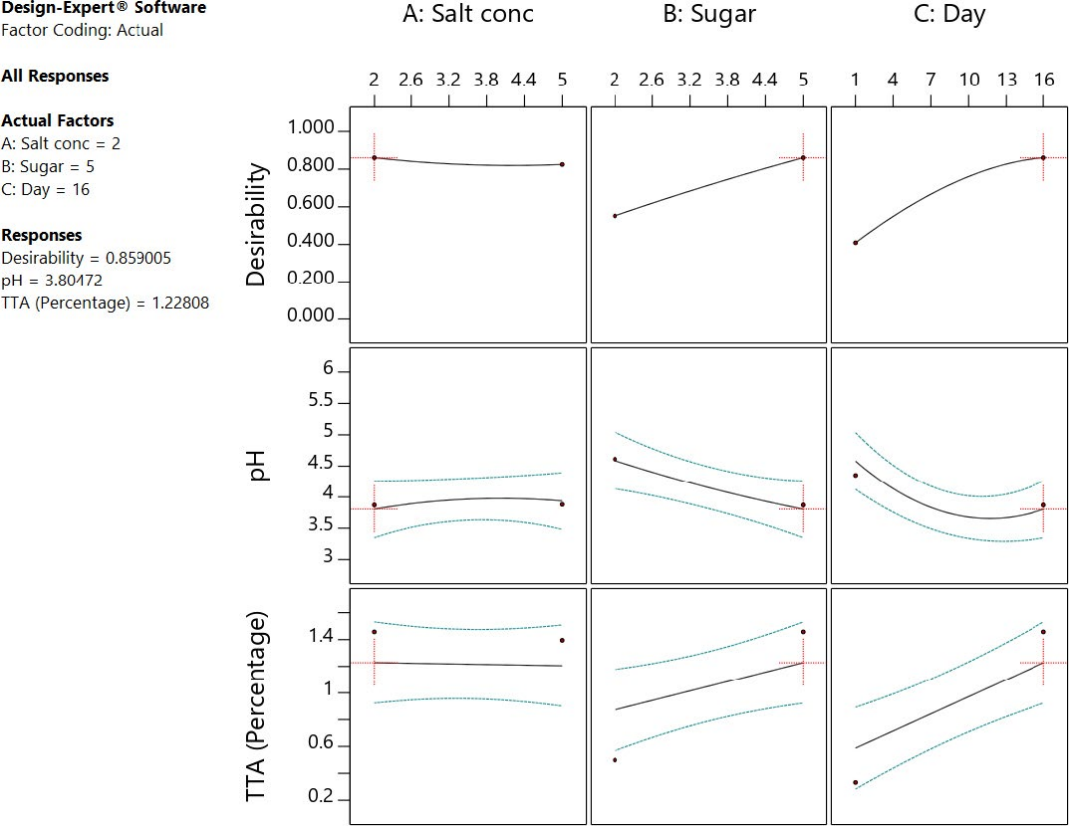
Optimized fermentation parameters for production of soured cowpea leaves

The individual factors of period of fermentation and sugar significantly (*p* < .05) affected both pH and titratable acidity as shown in Figures 5 and 6. Increasing the sugar concentration significantly (*p* < .05) increased both the pH and titratable acidity. Increasing salt concentration influenced the change in the pH, whereas there was no significant change in the titratable acidity. There was no interaction between the factors to influence titratable acidity. On the other hand, the three factors had interactions to influence the pH of the fermented cowpea leaves as shown in Figures 7 and 8. The optimal points for fermentation parameters were determined as salt concentration of 2%, sugar concentration of 5%, and a period of fermentation of 16 days. The optimal response parameters were found to be a pH of 3.8 and titratable acidity of 1.23%; the desirability of the solution generated was 0.859 (Figure 9). The validation of the response variables of the optimally fermented cowpea leaves yielded pH of 3.75 ± 0.07 and titratable acidity of 1.22 ± 0.01%.

### 3.3 Characterization of LAB isolates in the fermentation of cowpea leaves

**FIGURE 10:**
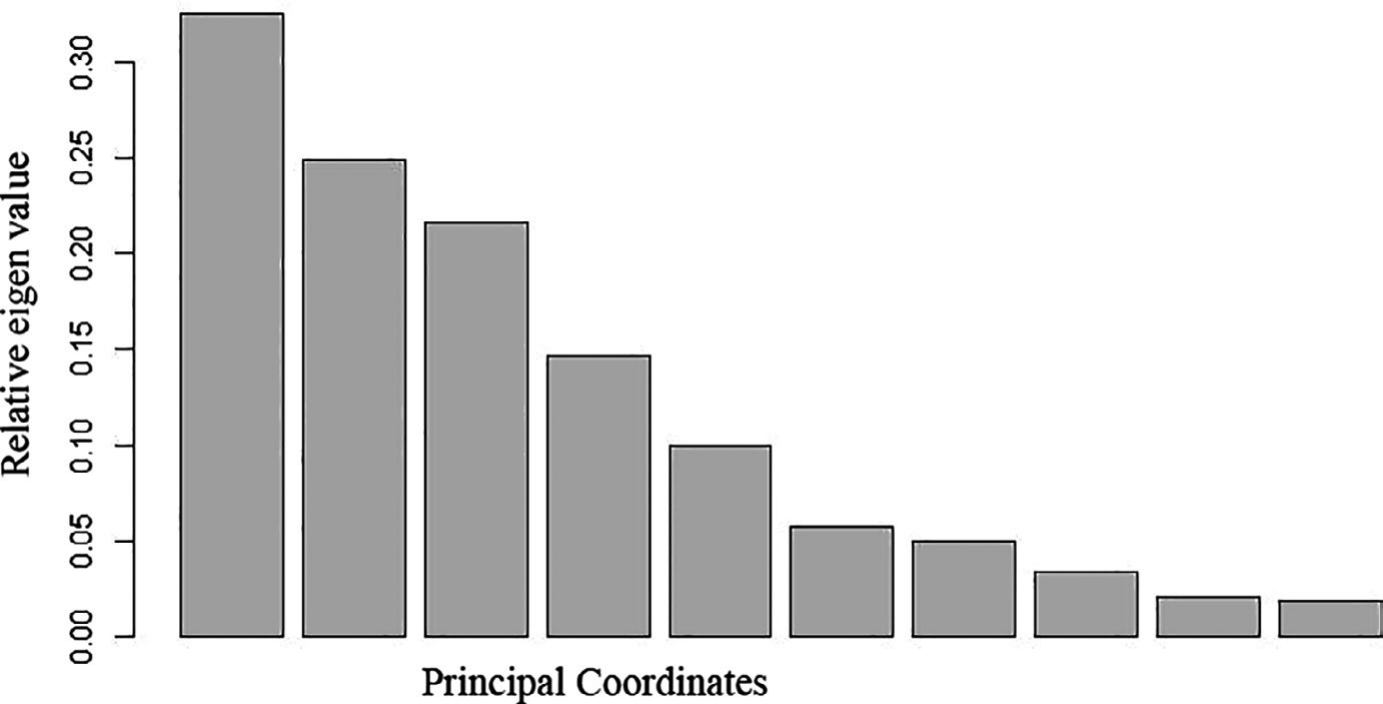
Relative eigen values explaining variation in the biochemical traits of lactic acid bacteria isolates from fermentation of cowpea leaves

**FIGURE 11:**
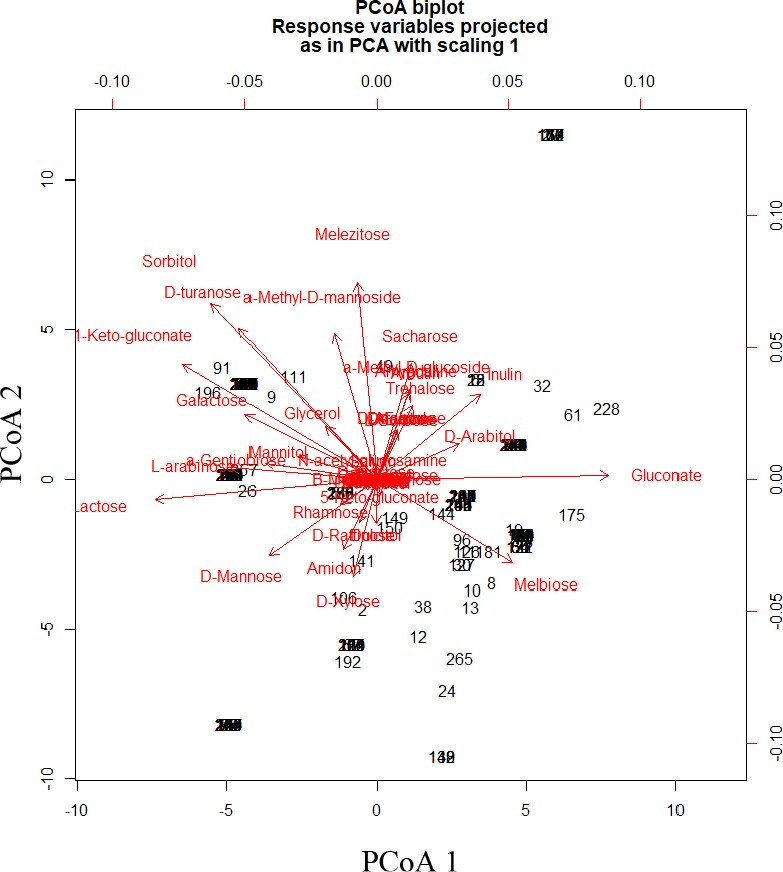
Principal coordinate analysis of biochemical traits of lactic acid bacteria isolates from spontaneous fermentation of cowpea leaves. PCoA 1‐First principal coordinate, PCoA 2‐Second principal coordinate

**FIGURE 12:**
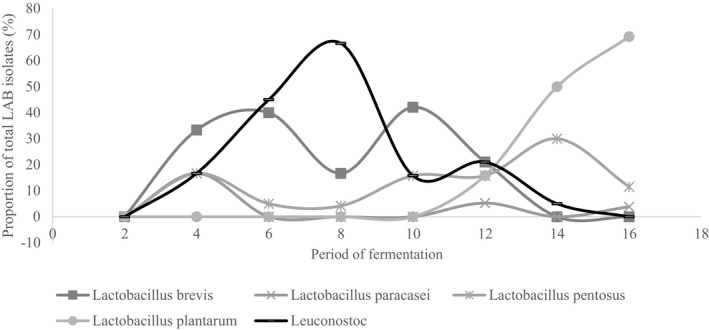
Dominant lactic acid bacteria involved in optimized fermentation of cowpea leaves

**FIGURE 13:**
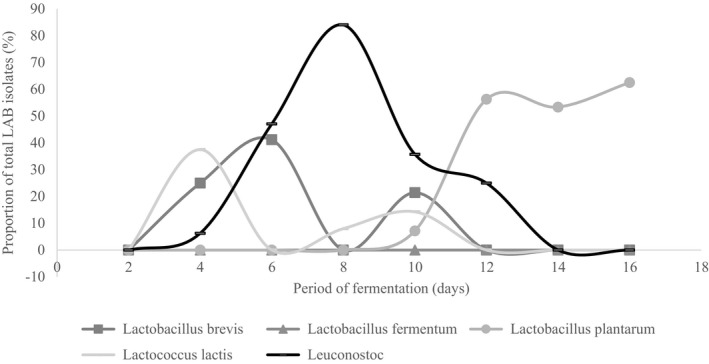
Dominant lactic acid bacteria involved in spontaneous fermentation of cowpea leaves

The optimally fermented cowpea leaves had a significantly (*p* < .05) lower pH of 3.8 ± 0.11 and significantly (*p* < .05) higher titratable acidity of 1.22 ± 0.33% lactic acid than that of spontaneously fermented leaves, pH and titratable acidity 4.0 ± 0.1 and 0.99 ± 0.07% lactic acid, respectively. The first two principal coordinates explained 57.4% variation in the biochemical traits of the microbial isolates (Figure 10). Thirteen different clusters of LAB isolates were formed based on their biochemical characterization that were reduced to thirteen definitive variable traits (Figure 11). Thirteen different LAB cultures were identified with the dominant ones being genus *Leuconostoc* (74), *Lactobacillus plantarum* (64), *Lactobacillus brevis* (42), and *Lactobacillus pentosus* (34) as shown in Appendix S1. Fermentation in both spontaneous and optimized processes was divided into three distinct stages based on microbial dominance: initial stage, intermediate stage, and final stage. In the initial stage of spontaneous fermentation, the dominant species were *L. brevis* and *L. lactis*, whereas only *L. brevis* dominated the initial stage of the optimized process (Figures 12 and 13). The genus *Leuconostoc* and species *L. plantarum* were the dominant LAB in both spontaneous and optimized processes at the intermediate and the final stages, respectively.

## 4 DISCUSSION

### 4.1 Model fitting for optimization of fermentative parameters

The fitted model revealed that the fermentation parameters (salt and sugar concentrations and period of fermentation) were of significance in influencing the pH and titratable acidity as response variables in fermentation process. The models accounted for 89% variation and 60% variation in the pH and titratable acidity, respectively. The investigative independent variables therefore were good predictors of the response variables owing to the high variation they account for. This is further emphasized by the finding of a good relationship between the actual and predicted values (Bai et al., 2014); qualifying the assumptions of the study that sugar and salt concentrations and period of fermentation influence fermentative action of vegetables. In the current study, pH and titratable acidity were used as proxy indicators of microbial activity in the fermentation process. The utilization of the RSM model is aimed at achieving the optimal points with limited resources and time (Jaiswal et al., 2012). The linear factors significantly affected the pH and the titratable acidity, whereas in the case of pH, there was an added effect of the quadratic factor. Fermentation of the vegetable induces increasing acidity, lowers pH, and increases titratable acidity, in the vegetables over increasing duration of fermentation. The effect of this is prolongation of the shelf‐life while improving on the sensory profile of the product. Considering the high postharvest losses in the cowpea leaves value chain, Wafula et al. (2016) recommend the utilization of fermentation for prolonged shelf‐life of the vegetable products.

Whereas the interaction coefficient did not show significant effect in predicting the model, the quadratic coefficient of the period of fermentation significantly influenced the rate of fermentative microbial activity. The implication of this is that the optimal parameters of the independent variables (sugar and salt concentrations and period of fermentation) are not within the extremes but rather on the response surface. The relationship between the predictor and response variables can therefore not easily be revealed through a hyperplane but rather a 3‐dimensional response surface curve.

### 4.2 Effect of sugar and salt concentrations and period of fermentation on pH and titratable acidity of soured cowpea leaves

Concentrations of sugar and salt and the period of fermentation have been independently established as factors that influence the fermentation process of vegetables (Muchoki et al., 2010; Xiong et al., 2016). This study found that with increasing sugar concentration, the pH and titratable acidity reduced and increased, respectively. Similarly, the period of fermentation was also of influence on the pH and titratable acidity. However, this only holds as a fact until the fermentable sugars are totally depleted (Muchoki, 2007). Yang et al. (2020) reported that the progressive increase in pH and titratable acidy in vegetable fermentation eventually cease due to the depletion of the available sugar in the fermentative solution. Similarly in another study by Kasangi et al. (2010), it was reported that notwithstanding the concentration of sugar utilized in the fermentation process, the progression curve for fermentation flattened after sometime indicative of depletion of fermentative sugars, the substrate utilizable in fermentation. Additionally, it is documented that sugar addition had an effect on the sensory quality of the soured vegetables, therefore the need for such optimization (Sui et al., 2019).

Increasing the salt concentration did not result in any increase in the titratable acidity nor decrease in the pH. In their study, Ziadi et al. (2019) reported the need to optimize salt concentrations utilized in lactic acid fermentation of vegetables for pH did not significantly change with changing concentrations of the brine. On the other hand, Muchoki (2007) reported that increasing salt concentrations in the fermentation of cowpea leaves would result in decreasing pH and increasing titratable acidity. However, in the latter study, it was also observed that increasing the salt concentrations above 2% (w/v) would inhibit growth of the fermentative bacteria.

The current study found that in order to produce optimally soured cowpea leaves, sugar concentration of 5% (w/v), salt concentration of 2% (w/v), and a fermentation period of 16 days have to be observed. Kasangi et al. (2010) utilized fermentable sugars to the tune of 3% in his optimization trials; however, the attained titratable acidity of 0.6% is lower than the optimal points obtained in this study. This was also a demerit that was realizable in the study by Muchoki (2007), whereby the target 1.5% for the titratable acidity was not met. Jagannath et al. (2012) explain these occurring phenomena in vegetable fermentation by elucidating the occurrence of osmotic stress with increasing levels of the fermentation parameters, salt and sugar concentrations; and depletion of the substrate utilizable over lengthened period of fermentation.

### 4.3 Biochemical characterization of fermentative bacteria involved in the production of soured cowpea leaves

The attained acidity in the optimally fermented leaves falls below the threshold for classification of high acid foods of pH less than 4.6 (Cunningham, 2009). The high acidity realizable in the optimally fermented product depictive of more improved microbial activity than the spontaneous process. The major fermentative bacteria associated with the production of soured vegetables products include *Lactobacillus acidophilus*, *Lactobacillus fermentum*, *Lactococcus lactis*, *Leuconostoc mesenteroides,* and *Lactobacillus plantarum* (Wafula et al., 2016). Similar LAB isolates were found to dominate the fermentation of cowpea leaves in the current study. It has been hypothesized that the addition of sugar in vegetables, known to be low in sugars, fastens the transition from homofermentation to heterofermentation. Zhao et al. (2019) reported a slow transition of homofermentation to heterofermentation which he solved by addition of molasses. In the current study, the fermentation in spontaneous process was originally dominated by the homofermenter (*L. lactis*) before the obligate heterofermenter (*L. brevis* and genus *Leuconostoc*) dominated the intermediate stage. With the addition of sugar, the transition to heterofermentation is faster, hence the domination of *L. brevis* of the onset of the process in the optimized process. Similarly, in cabbages that had 2.8% glucose, 1.5% fructose, and 0.3% sucrose, Jagannath et al.  (2012) found domination of the cabbage fermentation by obligate heterofermenters. In both the spontaneous and optimized fermentation processes, there was a succession of the *Leuconostoc spp*. by the *L. plantarum*. Similar findings were reported by Szutowska and Gwiazdowska (2020) in their study on kales where the *Leuconostoc spp*. was succeeded in domination of the fermentation process by predominantly the *L. plantarum*.

*L. plantarum* is a facultative homofermenter, implying that it largely produces lactic acid as the product of fermentation; however, it can also degrade pentoses (C_5_ Sugars) such as the xylose to produce lactic acid, acetic acid, and alcohol. Both in spontaneous and optimized fermentation, the process was predominated by *L. plantarum* in the final stage of fermentation (Ashaolu & Reale, 2020). Dominance of the facultative homofermenter, *L. plantarum*, was for a longer period in spontaneous fermentation than in optimized process. The microbe has displayed capacity to metabolize both hexose and pentose sugars; this catabolic flexibility has contributed to its dominance in food fermentation processes (Filannino et al., 2014). The microorganism has been found to improve the antioxidant activity of fermented vegetables while minimizing deterioration of microbial quality. There is, however, need to investigate the possibility of any antagonism between *L. plantarum* and the dominant LAB cultures such as *Leuconostoc spp*. and *L. brevis* in the fermentation processes.

## 5 CONCLUSION

The optimization of the fermentation process of cowpea leaves in this study found that sugar and salt had to be added as ingredients and the period of fermentation observed at 16 days. From this study, cowpea leaves products were acidified (high acid food) with a low pH of 3.8 and titratable acidity of 1.22%. The optimization induced changes to the microbial profile of the fermentation process of the vegetables. The dominant LAB cultures were found to change in the onset stage of fermentation of cowpea leaves. Additionally, the domination of the *L. plantarum* in the microbial culture was found to be limited in the optimized process. The limitation of the current study was that the antagonism among the microbial species in the cultures was not investigated, and further recommendation is to have this evaluated for potential development of symbiotic starter cultures. Further research is also recommended to establish the sensory profiling and improvements occurring in the optimally fermented product in comparison with the spontaneously fermented leaves. This study contributes to the prospect of commercialization and standardization of quality and production process of fermented cowpea leaves for it provides inputs toward improving the low‐cost processing techniques currently being utilized among smallholder groups and households.

## CONFLICT OF INTEREST

None.

## Supporting information

Appendix S1

## Data Availability

Data available on request from the authors. The data that support the findings of this study are available from the corresponding author upon reasonable request.
