# Supplementary material for: Comparative profiling of lactic acid bacteria isolates in optimized and spontaneous fermentation of cowpea leaves
Source: Food Sci Nutr. 2021 Jan 23;9(3):1651–64. doi: 10.1002/fsn3.2140 (PMC7958546; doi:10.1002/fsn3.2140)
Supplement: Supplementary file 1 — Appendix S1 [file FSN3-9-1651-s001.docx]

## Appendix 1: Lactic Acid Bacteria Isolates involved in the fermentation of cowpea leaves

| **Carbohydrate** | **Microbial isolates** | | | | | | | | | | | | |
| --- | --- | --- | --- | --- | --- | --- | --- | --- | --- | --- | --- | --- | --- |
|  | **A** | **B** | **C** | **D** | **E** | **F** | **G** | **H** | **I** | **J** | **K** | **L** | **J** |
| Control | -ve | -ve | -ve | -ve | -ve | -ve | -ve | -ve | -ve | -ve | -ve | -ve | -ve |
| Glycerol | -ve | +ve | +ve | -ve | -ve | -ve | -ve | -ve | -ve | -ve | -ve | -ve | -ve |
| Erythritol | -ve | -ve | -ve | -ve | -ve | -ve | -ve | -ve | -ve | -ve | -ve | -ve | -ve |
| D-Arabinose | -ve | +ve | -ve | -ve | -ve | -ve | -ve | -ve | -ve | -ve | -ve | -ve | -ve |
| L-arabinose | +ve | -ve | +ve | +ve | +ve | -ve | +ve | +ve | -ve | +ve | +ve | +ve | +ve |
| Ribose | +ve | +ve | +ve | +ve | +ve | +ve | +ve | +ve | -ve | +ve | +ve | +ve | +ve |
| D-Xylose | +ve | -ve | +ve | -ve | +ve | -ve | +ve | +ve | -ve | +ve | +ve | +ve | -ve |
| L-Xylose | -ve | -ve | -ve | -ve | -ve | -ve | -ve | -ve | -ve | -ve | -ve | -ve | -ve |
| Adonitol | -ve | -ve | -ve | -ve | -ve | -ve | -ve | -ve | -ve | -ve | -ve | -ve | -ve |
| B-Methyl-D-Xylose | -ve | -ve | -ve | -ve | -ve | -ve | -ve | -ve | -ve | -ve | -ve | -ve | -ve |
| Galactose | +ve | +ve | +ve | +ve | +ve | -ve | +ve | +ve | -ve | +ve | -ve | +ve | +ve |
| D-Glucose | +ve | +ve | +ve | +ve | +ve | +ve | +ve | +ve | -ve | +ve | +ve | +ve | +ve |
| D-Fructose | +ve | +ve | +ve | +ve | +ve | +ve | +ve | +ve | -ve | +ve | +ve | +ve | +ve |
| D-Mannose | -ve | -ve | +ve | +ve | +ve | +ve | -ve | +ve | -ve | +ve | +ve | +ve | +ve |
| L-Sorbose | -ve | +ve | -ve | -ve | -ve | -ve | -ve | -ve | -ve | -ve | -ve | -ve | -ve |
| Rhamnose | -ve | -ve | +ve | -ve | -ve | -ve | +ve | -ve | -ve | -ve | +ve | -ve | -ve |
| Dulcitol | -ve | -ve | -ve | -ve | -ve | -ve | -ve | -ve | +ve | +ve | +ve | -ve | -ve |
| Inositol | -ve | +ve | -ve | -ve | -ve | -ve | -ve | -ve | -ve | -ve | -ve | -ve | -ve |
| Mannitol | -ve | +ve | +ve | +ve | +ve | +ve | +ve | +ve | -ve | -ve | -ve | +ve | -ve |
| Sorbitol | +ve | +ve | +ve | +ve | -ve | -ve | -ve | -ve | -ve | -ve | -ve | -ve | -ve |
| a-Methyl-D-mannoside | +ve | -ve | -ve | +ve | -ve | -ve | -ve | -ve | -ve | -ve | -ve | -ve | +ve |
| a-Methyl-D-glucoside | +ve | +ve | +ve | +ve | -ve | +ve | +ve | -ve | +ve | +ve | +ve | -ve | +ve |
| N-acetyl-glucosamine | +ve | +ve | +ve | +ve | +ve | +ve | +ve | +ve | +ve | +ve | +ve | +ve | +ve |
| Amygdaline | +ve | +ve | +ve | +ve | -ve | +ve | +ve | -ve | +ve | +ve | +ve | -ve | +ve |
| Arbutin | +ve | +ve | +ve | +ve | -ve | +ve | +ve | -ve | +ve | +ve | +ve | -ve | +ve |
| Esculin | +ve | +ve | +ve | +ve | +ve | +ve | +ve | +ve | +ve | +ve | +ve | +ve | +ve |
| Salicin | +ve | +ve | +ve | +ve | +ve | +ve | +ve | +ve | +ve | +ve | +ve | +ve | +ve |
| Cellobiose | +ve | +ve | +ve | +ve | +ve | +ve | +ve | +ve | -ve | +ve | +ve | +ve | +ve |
| Maltose | +ve | +ve | +ve | +ve | +ve | +ve | +ve | +ve | +ve | +ve | +ve | +ve | +ve |
| Lactose | -ve | -ve | +ve | +ve | +ve | -ve | -ve | +ve | +ve | +ve | -ve | +ve | +ve |
| Melbiose | +ve | -ve | +ve | -ve | -ve | +ve | +ve | -ve | -ve | +ve | +ve | -ve | -ve |
| Sacharose | +ve | +ve | +ve | +ve | -ve | +ve | +ve | -ve | -ve | -ve | -ve | -ve | +ve |
| Trehalose | +ve | +ve | +ve | +ve | -ve | +ve | +ve | -ve | -ve | +ve | +ve | -ve | +ve |
| Inulin | +ve | +ve | -ve | -ve | -ve | -ve | -ve | -ve | -ve | -ve | -ve | -ve | -ve |
| Melezitose | +ve | +ve | -ve | +ve | -ve | -ve | -ve | -ve | -ve | -ve | -ve | -ve | -ve |
| D-Raffinose | +ve | -ve | +ve | +ve | +ve | +ve | +ve | +ve | -ve | +ve | +ve | +ve | -ve |
| Amidon | -ve | -ve | -ve | -ve | +ve | -ve | -ve | +ve | -ve | -ve | -ve | +ve | -ve |
| Glycogen | -ve | -ve | -ve | -ve | -ve | -ve | -ve | -ve | -ve | -ve | -ve | -ve | -ve |
| Xylitol | -ve | -ve | -ve | -ve | -ve | -ve | -ve | -ve | -ve | -ve | -ve | -ve | -ve |
| a-Gentiobiose | +ve | -ve | +ve | +ve | +ve | -ve | +ve | +ve | +ve | +ve | +ve | +ve | +ve |
| D-turanose | -ve | +ve | +ve | +ve | -ve | -ve | +ve | -ve | -ve | -ve | -ve | -ve | -ve |
| D-Lyxose | -ve | +ve | -ve | -ve | -ve | -ve | -ve | -ve | -ve | -ve | -ve | -ve | -ve |
| D-Tagatose | -ve | +ve | -ve | -ve | -ve | -ve | -ve | -ve | +ve | +ve | +ve | -ve | +ve |
| D-Fucose | -ve | +ve | -ve | -ve | -ve | -ve | -ve | -ve | -ve | -ve | -ve | -ve | -ve |
| L-Fucose | -ve | -ve | -ve | -ve | -ve | -ve | -ve | -ve | -ve | -ve | -ve | -ve | -ve |
| D-Arabitol | +ve | -ve | -ve | -ve | -ve | -ve | -ve | -ve | -ve | -ve | -ve | -ve | -ve |
| L-Arabitol | -ve | -ve | -ve | -ve | -ve | -ve | -ve | -ve | -ve | -ve | -ve | -ve | -ve |
| Gluconate | +ve | +ve | -ve | -ve | -ve | +ve | +ve | -ve | -ve | -ve | -ve | -ve | -ve |
| 1-Keto-gluconate | -ve | -ve | +ve | +ve | -ve | -ve | -ve | -ve | -ve | -ve | -ve | -ve | -ve |
| 5-Keto-gluconate | -ve | -ve | -ve | -ve | -ve | -ve | -ve | -ve | -ve | -ve | -ve | -ve | -ve |
| Number of of isolates | 1 | 42 | 3 | 8 | 34 | 64 | 1 | 16 | 74 | 3 | 2 | 13 | 6 |

-ve gave a positive test whereas –ve gave a negative test. A-*Lactobacillus acidophilus*, B-*Lactobacillus brevis*, C-*Lactobacillus fermentum*, D-*Lactobacillus paracasei*, E-*Lactobacillus pentosus*, F-*Lactobacillus plantarum*, G-*Lactobacillus spp*, E-*Lactococcus lactis*, F-*Leuconostoc*, G-*Leuconostoc dextranicum*, H-*Pediococcus cerevisiae*, I-*Pediococcus pentosaceus* and J-*Pediococcus spp*
